# Modulators of MicroRNA Function in the Immune System

**DOI:** 10.3390/ijms21072357

**Published:** 2020-03-29

**Authors:** Yunhui Jia, Yuanyuan Wei

**Affiliations:** 1 Department of Immunology, School of Basic Medical Sciences, Fudan University, Shanghai 200032, China; 2 Department of Immunology, Shanghai Key laboratory of Bioactive Small Molecules, State Key Laboratory of Medical Neurobiology, School of Basic Medical Sciences, Fudan University, Shanghai 200032, China

**Keywords:** microRNA biogenesis, immune cells, miRNA site, exosome, macrophage

## Abstract

MicroRNAs (miRNAs) play a key role in fine-tuning host immune homeostasis and responses through the negative regulation of mRNA stability and translation. The pathways regulated by miRNAs are well characterized, but the precise mechanisms that control the miRNA-mediated regulation of gene expression during immune cell-development and immune responses to invading pathogens are incompletely understood. Context-specific interactions of miRNAs with other RNA species or proteins may modulate the function of a given miRNA. Dysregulation of miRNA function is associated with various human diseases, such as cardiovascular diseases and cancers. Here, we review the potential modulators of miRNA function in the immune system, including the transcription regulators of miRNA genes, miRNA-processing enzymes, factors affecting miRNA targeting, and intercellular communication.

## 1. Introduction

The development and function of immune cells are vital for host defense against external and internal threats and for immune resolution following the elimination of threats, which requires rapid changes in the transcriptome and proteome. These changes are tightly regulated at both the transcriptional and post-transcriptional levels, where microRNAs (miRNAs) have been demonstrated to be crucial molecular players. miRNAs are small non-coding RNAs (about 18–22 nucleotides in length) produced by a multi-step process involving a series of enzymes and proteins [1]. The genes encoding miRNAs are transcribed by either RNA polymerase II or III as primary miRNAs (pri-miRNAs) containing a cap structure at the 5′ end and polyadenylation at the 3′ end [2]. The nuclear microprocessor complex, composed of the ribonuclease (RNase) III enzyme Drosha and the RNA-binding protein DGCR8, processes pri-miRNAs into the precursor miRNAs (pre-miRNAs) which are exported into the cytoplasm facilitated by Exportin 5 and the GTP-binding nuclear binding protein RAN•GTP [3,4,5,6,7]. RNase III enzyme Dicer then cleaves pre-miRNAs into mature miRNA duplexes through binding to the two-nucleotide overhang at their 3′ end generated by Drosha [8,9]. One strand of the miRNA duplex is usually incorporated into the miRNA-induced silencing complexes (mRISCs) through the Argonaut (Ago) proteins, which guide the binding of miRNAs to complementary sites mainly located in the 3′ untranslated regions (3′ UTRs) of the target mRNAs [10,11]. miRNAs are estimated to control 30%–80% of mammalian genes by repressing translation and reducing the stability of target mRNAs [12,13,14].

The immunoregulatory role of miRNAs was first implicated by conditional ablation of key components of the miRNA biogenesis pathway, such as *Drosha*, *Dgcr8*, and *Dicer*, in various immune cells at distinct stages of immune responses [15,16,17,18,19,20,21]. As a matter of course, a large number of studies subsequently investigated the immune function and downstream targets of individual miRNAs, normally using gain- and loss-of-function approaches, which have been thoroughly reviewed elsewhere [22,23,24]. However, considering that substantial mammalian mRNAs are miRNA targets [13] and that the miRNA machinery is extremely complicated, it is critical to understand how miRNA biogenesis and function are tightly regulated in the immune system, despite the currently limited number of studies. Modulation of miRNA-mediated gene regulation may take place in multiple steps, including miRNA transcription, miRNA biogenesis and turnover, and target selection [11,25,26]. This review summarizes our initial knowledge of the mechanisms applied by the immune cells to accurately outline miRNA function, including the recruitment of transcription factors, the modification of enzymes involved in miRNA biogenesis, changes in the variants of miRNA target sites, and the uptake of exogenous miRNAs.

## 2. Regulation of miRNA Transcription

As non-coding RNAs, the function of miRNAs is largely based on their transcriptional expression levels, which exhibit tissue- and cell-specific patterns [27]. To respond to environmental challenges, the miRNA transcriptional scenario is coordinately regulated by transcription factors (TFs), chromatin modifications, and signaling molecules. TFs usually recognize small 6–12 bp-long degenerate DNA sequences that are organized into *cis*-regulatory modules, known as promoters and enhancers. In each cell type, only a small fraction of the *cis*-regulatory repertoire is actively associated with lineage-determining TFs and specific chromatin modifications, which are used to control gene expression determined by the upstream signaling molecules [28]. While the precise locations of *cis*-regulatory modules have not yet been characterized for most miRNAs [29], it has been clear that both TFs and chromatin modifications contribute to miRNA gene transcription in immune cells [11,25,26,30].

### 2.1. TFs of miRNA Genes in Myeloid Cells

Myeloid cells, including monocytes/macrophages, dendritic cells (DCs), granulocytes, and mast cells, are all derived from hematopoietic progenitors in bone marrow. Lineage-specific TFs, such as PU.1, are essential to determine myeloid cell diversity and functional specificity during differentiation [28,31,32]. PU.1 has been shown to control the transcription of several hematopoietic miRNAs, such as miR-223, miR-23a and miR-142-3p (Table 1) [33,34,35,36]. miR-223 expression is steadily upregulated during granulocyte differentiation, and it plays a central role in fine-tuning granulocyte differentiation and activation: The deletion of *Mir223* led to an expanded granulocytic compartment, neutrophil hypermaturation, and hyperactivity, and the development of inflammatory lung pathology upon endotoxin challenge in mice [34]. Similarly, transcription of the miR-23a cluster facilitated hematopoietic stem cell differentiation into myeloid cells at the expense of B cells [36,37]; and miR-142-3p is a crucial negative regulator of Interleukin 6 (IL-6) in both dendritic cells [38] and macrophages [39] through direct targeting.

Myeloid cells are largely responsible for the innate immune response and recognize invading pathogens through germline-encoded pattern recognition receptors (PRRs), such as toll-like receptors, that sense pathogen-associated molecular patterns (PAMPs) expressed by microorganisms, such as lipopolysaccharide (LPS) [40,41]. Signaling from PRRs activates specific transcriptional networks, which lead to different myeloid cell activations and, eventually, distinct effector functions [42]. Nuclear factor-κB (NF-κB) is the central transcription factor that orchestrates the inflammatory activation of myeloid cells [43] and has been shown to control transcription of several miRNA genes (Table 1). NF-κB is able to bind to the promoters of both miR-146a and miR-155, which were upregulated in THP-1 monocytes challenged by LPS [44,45,46,47,48]. While miR-146a targets the IL-1 receptor associated kinase 1 (IRAK1) and TNF receptor-associated factor 6 (TRAF6) to inhibit the inflammatory response [44], miR-155 expression is required for inflammatory macrophage activation [47,49]. In addition to NF-κB, the transcription factor E26 avian leukemia oncogene 2 (Ets2) is indispensable for the induction of miR-155 in macrophages by LPS (Table 1) [47]. Moreover, NF-κB is required for *Mycobacterium tuberculosis* (Mtb)-induced miR-33 and miR-33* expression in macrophages, by which Mtb inhibits host autophagy, lysosomal function, and fatty acid metabolism to support its own survival [50]. Smoking or toll-like receptor ligands upregulated miR-22 expression, depending on the binding of NF-κB to the miR-22 host gene, which is required for DC activation through miR-22-mediated targeting of the histone deacetylase HDAC4 and the subsequent activation of transcription factor AP-1 [51]. *Mir22* deficient mice exhibited impaired Th17 responses and failed to develop pulmonary emphysema after exposure to smoke or nanoparticulate carbon black, which was probably due to impaired DC activation.

### 2.2. TFs of miRNA Genes in T Cells

T lymphocytes are the core components in the adaptive immune system, and as with myeloid cells, originate from bone marrow progenitors, which migrate to the thymus for maturation and selection into the CD4^+^ or CD8^+^ lineages, and are subsequently exported to the periphery. Peripheral T cells comprise different subsets, including naive T cells, which differentiate into distinct effector subsets that produce specialized cytokines against a variety of pathogenic challenges [59]. 

Naive CD4^+^ T cells differentiate into distinct effector subsets through the activity of different TFs, such as T-bet for T helper 1 (Th1) cells [60], GATA binding protein 3 (GATA-3) for Th2 cells [61], RORγt for Th17 cells [62], and forkhead box P3 (Foxp3) for regulatory T cells (Treg cells) [63]. Foxp3+ Treg cells constitute a unique T cell lineage that is essential for the prevention of self-destructive immune responses [64,65,66]. Foxp3 is able to bind to an intron region within the host gene of miR-155, Bic, and is required for the maintenance of high expressions of miR-155 in Treg cells (Table 1) [52,65,67,68]. Both the number and proliferative potential of Treg cells were impaired in mice deficient in *Mir155* [52].

In opposition to TFs, the transcription repressor B cell leukemia/lymphoma 6 (Bcl-6) determines the follicular helper T (Tfh) cell lineage by suppressing RORγt and T-bet, and several miRNAs, including miR-17~92 cluster of miRNAs, which are transcribed as a polycistronic primary transcript encoding six different miRNAs (Table 1) [53]. High expressions of Bcl-6 may lead to the downregulation of two members of the miR-17~92 cluster, miR-17 and miR-20a, which contribute to the induction of the hallmark molecules C-X-C motif chemokine receptor 5 (CXCR5, a chemokine receptor essential for the migration of CD4^+^ T cells to B cell follicles) in Tfh cells [53]. In addition to this cell-intrinsic signaling, another study showed that the miR-17~92 family is a positive regulator of Tfh development by regulating the inducible costimulatory ICOS signaling, which controls the follicular recruitment of CD4^+^ T cells, depending on the ICOS ligand expression by follicular bystander B cells, rather than on CXCR5 and Bcl-6 expressions [69,70]. Nevertheless, inducible miR-17~92 family transcription is necessary for Tfh cell development and function. 

Moreover, naive CD8^+^ T cells proliferate and differentiate into a variety of effector and memory cell types upon an antigen encounter. Cytotoxic effector cells are responsible for controlling and eventually eliminating pathogens, while memory T cells are differentiated from a small fraction of effector T cells that survive following pathogen clearance. CD8^+^ T cell exhaustion, characterized by a loss in effector function, was identified in chronic viral, bacterial, and parasitic infections as well as in human cancers [71]. All these events are regulated by signal-driven cell-type-specific transcriptional responses [72]. The TF nuclear factor of activated T cells (NFAT) is a key regulator of T cell activation and exhaustion of activated CD8^+^ T cells [73,74]. The binding of NFAT was observed upstream of the miR-31 coding gene in only activated, but not in native, CD8^+^ T cells (Table 1) [54]. The induced expression of miR-31 caused the exhaustion of CD8^+^ T cells by enhancing the expression of multiple inhibitory molecules in chronic viral infections.

### 2.3. Epigenetic Modifications of miRNA Genes

It has become clear that epigenetic modifications at genic loci, such as histone modification and DNA methylation, cooperating with transcription factors, play a critical role in orchestrating the transcriptional changes associated with immune cell activation [75,76,77]. While the impact of alterations in the epigenetic landscape on miRNA expression has not been extensively investigated in immune cells, limited studies have implicated a regulatory role of epigenetic modification in miRNA expression during immune responses (Table 1). miR-495 transcription was repressed due to the methylation of its promoter in alveolar macrophages, stimulated with LPS, thereby contributing to inflammasome activation through the release of its direct target NLRP3 [55]. A study showed that NF-κB-dependent miR-146a transcription might be a result of impaired histone lysine demethylase Jumonji domain containing-3 (JMJD3) binding to the miR-146a promoter: JMJD3 demethylated H3K27me3 on the promoter of miR-146a and negatively regulates miR-146a transcription in macrophages, which was inhibited by NF-κB [56]. Conversely, the NF-κB binding capacity to the miR-146a promoter was inhibited by DNA methyltransferase and histone deacetylase in aged macrophages [78]. These findings suggest that miR-146a transcription is coordinated by both epigenetic modifications and TFs in activated macrophages. Additionally, higher DNA methylation in the VMP1/MIR21 locus was associated with lower expressions of mature miR-21 in CD4^+^ T cells [57]. In contrast, histone H3 acetylation of host genes encoding miRNAs, such as miR-155 and miR-181b, promoted the transcription of these miRNAs, thereby silencing the genes involved in class-switch DNA recombination and somatic hypermutation of B cells [58]. 

Despite the fact that several studies have paid attention to the transcriptional regulation of miRNA expression, there is still substantial room for improvement, especially considering the significant gap between the number of studies on how miRNAs regulate TFs or epigenetic modifiers and on how TFs or epigenetic modifiers regulate miRNAs.

## 3. Regulation of miRNA Machinery

**Figure 1:**
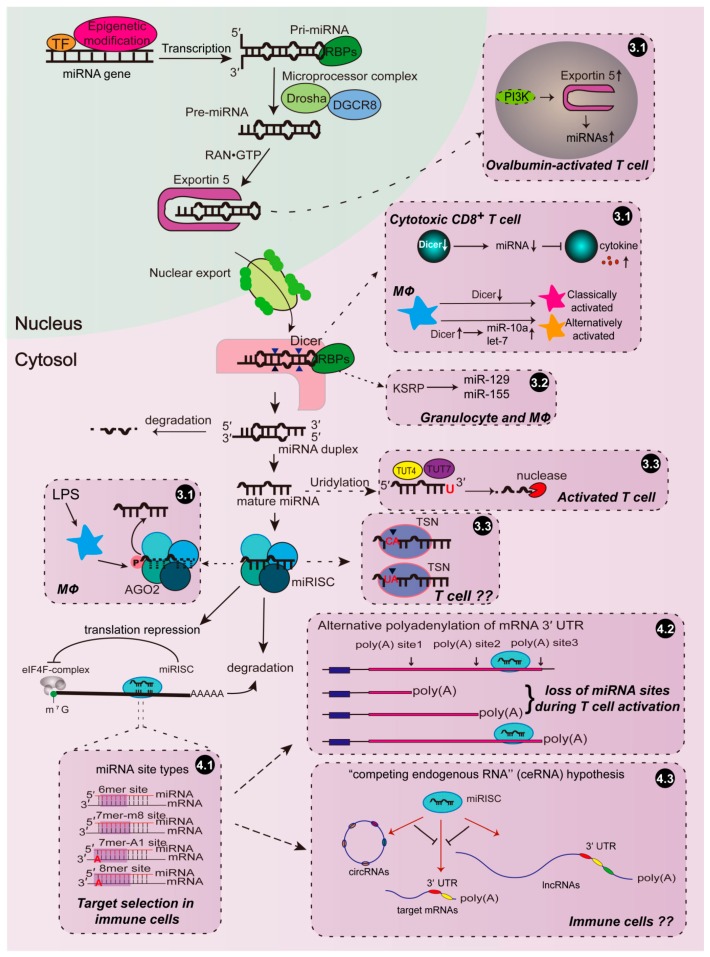
Regulation of microRNA (miRNA) function by modulating its biogenesis and targeting in the immune system. Immune cells orchestrate miRNA function through regulating or modifying the key enzymes and proteins involved in miRNA biogenesis, such as Exportin 5, Dicer, Ago2, and some RNA binding proteins (RBPs). Moreover, miRNAs may selectively target mRNAs according to the miRNA site types in distinct immune cells. miRNA-mediated targeting may also be regulated by competing endogenous RNA (ceRNAs) or affected by alternative poly(A) site usage in immune responses. The events in the miRNA biogenesis and targeting pathways that are able to be regulated during immune responses are summarized in the boxes outlined with dash lines and numbered according to the text. The arrows with solid lines indicate each step in the process of miRNA biogenesis, while the arrows with dash lines indicate how that step is modulated in immune cells.

Following transcription, the primary miRNA transcripts are processed by several steps until maturation and are subsequently loaded into RISCs to play effector functions, and modifications of components involved in the miRNA machinery can lead to rapid global or specific changes in miRNA abundance in immune cells (Figure 1).

### 3.1. Regulation of Conventional miRNA-Processing Enzymes and Proteins

Drosha and Dicer are two central enzymes involved in miRNA biogenesis. It has been shown that Dicer expression is significantly downregulated in cytotoxic CD8^+^ T cells activated by inflammatory signals, leading to a reduction in the expression of five miRNAs (among those miR-139 and miR-150 were identified as the effector miRNAs), which seemed to be essential for increased cytokine expression in activated CD8^+^ T cells [79]. Consistent with the inhibitory effect of Dicer on the inflammatory activation of CD8^+^ T cells, CD4^+^ T cells deficient in *Dicer* preferentially express interferon-γ (IFN-γ), the effector cytokine of the Th1 lineage [80]. In macrophages, altered miRNA profiles, due to up- or downregulated Dicer expression, modulate macrophage polarization towards either alternatively or classically activated phenotype [20]. The anti-inflammatory role of Dicer in macrophages promotes tumor growth and limits atherosclerosis, probably through the let-7-dependent suppression of IFN-γ/STAT1 signaling and miR-10a-guided cellular metabolism reprogram towards fatty acid oxidation, respectively [19,20]. These findings indicate that the effect of an altered Dicer expression is eventually reflected by only a few specific downstream miRNAs, which raises a question as to the mechanisms determining Dicer’s specificity. Moreover, although the function of enzymes that are central to miRNA biogenesis has been investigated in the other immune events, such as hematopoietic cell differentiation and dendritic cell development, by using several knockout mouse models experimentally [16,81,82,83], it is still unknown whether immune cells reprogram miRNA profiles through modulating Drosha/Dicer expression to meet the demand for immune responses physically or pathologically in these processes. 

Exportin 5 is responsible for the nuclear export of pre-miRNAs. Overall, increased miRNA expression is observed in ovalbumin-activated T cells, associated with an increased Exportin 5 protein level, probably depending on phosphoinositide-3-kinase (PI3K) activation [84]. However, the detailed mechanism needs to be further investigated.

The mature miRNAs generated by Dicer are subsequently loaded onto the effector complex RISCs, which involves the central AGO proteins that can be modulated by numerous modifications. Ago2 phosphorylation at Tyr529, caused by LPS stimulation, promoted a relief of cytokine mRNAs from miRNA-mediated repression in macrophages due to reduced miRNA binding to RISCs [85]. However, the kinase responsible for this event is still unknown. Global downregulation of miRNAs was observed during T cell activation [86,87,88], which was as least partially the result of mTOR-dependent proteasomal degradation of Ago2 [88].

While RNAi machinery directly functions as an antiviral defense in nematodes, plants, and insects [89], several studies implicate the opposite scenario in mammals. Poly inosine:cytosine (I:C), a mimic of dsRNA that is a common byproduct of viral replication, triggers poly-ADP-ribosylation and inhibition of RISCs in host cells to release the miRNA-mediated inhibition of cytotoxicity-associated IFN-stimulated genes (ISGs) [90]. It seems that the suppression of the whole RNAi machinery is the anti-viral strategy of host cells; conversely, *Herpevirus saimiri* uses its noncoding RNAs to degrade the miRNAs in host T cells in a sequence-specific and binding-dependent manner, such as miR-27, thereby interfering with host-cell gene expression [91].

### 3.2. Regulation of RNA-Binding Proteins

In addition to the classical miRNA-processing enzymes and proteins, it is increasingly apparent that a number of RNA-binding proteins (RBPs) modulate miRNA biogenesis through binding to pri- or pre-miRNAs [92,93,94]. A proteomics-based pull-down study in cell lines, including T cells and B cells, identified ~180 RBPs as capable of interacting specifically with distinct pre-miRNAs [92]. Among those proteins, a zinc-finger-containing protein, ZC3H7A, was able to regulate miRNA biogenesis specifically by recognizing the sequences in the apical loops of miRNA hairpins. Interestingly, ZC3H7A expression was induced by LPS in primary murine macrophages [95], suggesting its potential role in inflammatory macrophage activation, which is likely mediated by regulating miRNA processing. However, the detailed function of these RBPs is still heavily investigated during immune responses. 

The single RNA binding protein KH-type splicing regulatory protein (KSRP) facilitates selected pri- and pre-miRNA recruitment into the Drosha- or Dicer-containing miRNA processing complex by interacting with the terminal loop (Figure 1) [96]. KSRP induced granulocyte maturation, rather than monocytopoiesis, by promoting miR-129 biogenesis [97]. In addition to regulating the miR-155 transcript as discussed above, Ruggiero et al. found that LPS dramatically promoted KSRP-mediated miR-155 maturation in macrophages [98]. However, this study showed that both KSRP and miR-155 limited inflammatory mediator expression in macrophages in response to LPS—in opposition to other studies in which miR-155 was indicated as a pro-inflammatory miRNA [47,49,99]. It seems that miR-155 could play both pro- and anti-inflammatory roles in a cell-dependent manner upon specific stimuli [49,100,101,102,103], and further research will elucidate its specific role in immune responses. In contrast to the in vitro study, *Ksrp* deficient mice displayed a reduced inflammatory response, characterized by a reduction in C-X-C motif chemokine ligand 1 (CXCL1), inducible nitric oxide synthase (iNOS) and tumor necrosis factor (TNF), and a reduced infiltration of monocytes and neutrophils using the model of inflammatory arthritis [104], implicating the complicated network regulated by KSRP in the immune system. 

### 3.3. Regulation of miRNA Stability

In addition to biogenesis, the rate of miRNA decay can mediate the rapid and selective remodeling of the miRNA repertoire of cells, thereby influencing the cell’s destiny and function. As a component in RISCs, the endonuclease Tudor-SN (TSN) regulates miRNA degradation through cleaving at CA and UA dinucleotides bonds that are located more than five nucleotides away from miRNA ends [105], implicating the sequence-specific recognition of TSN. While it has been shown that TSN is highly expressed in CD3^+^ T cells, but not in macrophages, in mouse lymphoid organs [106], the effect of TSN on miRNA degradation has not been discovered in the immune system. However, uridylation-mediated miRNA degradation has been observed during T-cell activation, implicating the potential immune function of miRNA turnover, although miRNA uridylation was globally diminished and was associated with the downregulation of closely homologous RNA terminal uridyltransferases (TUTs) family members, TUT4 and TUT7, after T-cell activation [107].

## 4. Regulation of miRNA Targeting

Mature miRNAs bind to the target sites that are frequently located in the 3′ UTR of mRNA in RISCs, causing translation repression or mRNA degradation. The evidence for cell- and context-dependent miRNA target selection has emerged.

### 4.1. miRNA Targeting Efficacy

One factor affecting the efficacy of miRNA-target interactions is the miRNA site type. It is notorious that the complementary base pairing between miRNA and the target site is imperfect in animals, accompanied by the existence of several miRNA site types, which share the perfect match between miRNA “seed region” (nucleotides 2–7) and the site within the 3′ UTR of target mRNA. Besides the 6 nt seed match (6mer site), most target sites also have either a match to miRNA nucleotide 8 (7mer-m8 site) or an A across from miRNA nucleotide 1 (7mer-A1 site), or both (8mer site) (Figure 1) [13,108]. In an evolutionary context, the presence of several types of miRNA–mRNA binding sites indicates their necessary, but diverse, roles in miRNA-mediated regulation, which is still unclear. Hsin et al. investigated the transcriptome-wide targeting and gene regulation by miR-155 in multiple immune cell types and demonstrated that the miR-155 sites that are shared by the cell types frequently have stronger seed matches (such as 8mer sites), whereas the cell-type-specific sites exhibit less extensive seed complementarity (such as 6mer sites) [109]. However, the factors that determine the cell context-dependent miRNA target pools are yet to be identified. This scenario might be dependent on the miRNA expression level since the weaker site types can only be regulated in the presence of higher miRNA abundance.

### 4.2. Alternative Polyadenylation of mRNA 3′ UTR

mRNAs with the same coding sequence often have tandem UTR isoforms, in which alternative polyadenylation (APA) at proximal or distal poly(A) sites yield shorter or longer 3′ UTRs, respectively [110,111]. Thereby, miRNA sites located in the alternatively included region are only present in the long 3′ UTR isoform, leading to the fact that a cell-type-specific shift in APA results in a corresponding shift in miRNA-mediated regulation. Analyses of the global effects of cellular contexts on miRNA targeting revealed that approximately 10% of predicted miRNA targeting was influenced by the differential usage of 3′ UTR isoforms in different cell types [112]. Similarly, in Hsin’s study, poly(A) sequencing in multiple immune cell types (dendritic cell, CD4^+^ T cell, B cell, and macrophage) indicated the widespread existence of alternative polyadenylation in huge numbers of genes, in which miR-155 targets were significantly enriched, although the authors claimed that APA had a limited contribution in context-specific miR-155 targeting [109]. The increase in the usage of shorter 3′ UTR isoforms has been proposed to be a potential mechanism to evade miRNA-mediated regulation in activated CD4^+^ T lymphocytes [113]. However, the extent to which changes in the polyadenylation site usage impacts mRNA and protein expression in activated T cells is still debatable [114]. Notably, vesicular stomatitis virus infection caused average 3′ UTR length shortening, especially for immune-related genes, implicating the loss of miRNA sites in macrophages during antiviral innate immune responses [115].

Dysregulated RNA 3′ end processing contributes to human diseases [116]. In multiple cancers, including adult T-cell leukemia/lymphoma, diffuse large B-cell lymphoma, and stomach adenocarcinoma, the presence of structural variations leads to shortening of programmed cell death ligand 1 (PD-L1) 3′ UTR, causing the marked elevation of aberrant PD-L1 transcripts due to enhanced mRNA stabilization [117]. The long 3′ UTR of PD-L1 harbors several potential miRNA binding sites, such as those for miR-34 and miR-200; therefore, relief of PD-L1 from miRNA-mediated inhibition might be one of the mechanisms responsible for its increased expression in cancers, although it was not investigated in this study.

These examples could represent just the tip of the iceberg, as the extent to which differential usage of 3′ UTR isoforms affect miRNA targeting in immune responses, and in immune diseases have not been investigated across the transcriptome.

### 4.3. Competing Endogenous RNA Theory

The “competing endogenous RNA” (ceRNA) hypothesis—that RNA molecules influence each other’s levels by competing for binding to limited pools of miRNAs—was first proposed by Salmena et al. in 2011 [118]. A growing body of evidence supports the idea that certain long noncoding RNAs (lncRNAs, non-coding RNA species longer than 200 nucleotides) and circular RNAs (circRNAs, non-coding RNAs with covalently closed-loop structures) contain large numbers of miRNA sites and may function as ceRNAs (Figure 1) [119,120,121]. However, the related studies in the immune system are just the starting point. By sequestering miRNAs, lncRNAs growth arrest-specific 5 (GAS5) [122], HIX003209 [123], and HOX transcription antisense RNA (HOTAIR) [124] promote macrophage inflammatory response, while NIFK-AS1 [125] inhibits alternative macrophage activation. Among these, lncRNAs, GAS5, and HOTAIR, as well as MIAT [126], were either induced by oxidized low-density lipoprotein (oxLDL) or upregulated in atherosclerotic plaques, indicating their potential association with atherosclerosis progression. Computational analysis also revealed that the circRNA/lncRNA-miRNA–mRNA network is probably involved in foam-cell formation and atherosclerosis [127]. However, more studies are required to provide a deeper mechanistic insight into how lncRNAs and circRNAs compete with mRNAs for miRNA binding during immune responses and in immune diseases, probably depending on the miRNA site type.

## 5. Intercellular Communication

**Figure 2:**
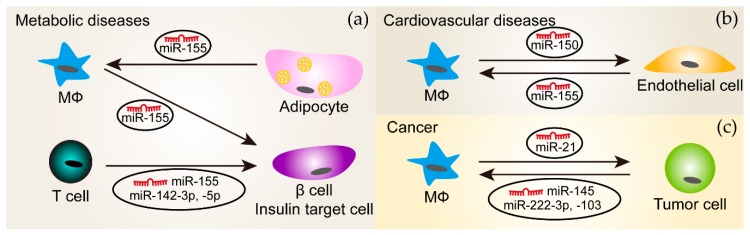
The delivery of miRNAs between immune cells and the other cell types through extracellular vesicles (EVs) in diseases. (**a**) In metabolic diseases (such as obesity and diabetes), miR-155 is transported from macrophages (MΦ) and T cells to β cells and insulin target cell types, respectively, which was shown to affect glucose metabolism. Moreover, EVs derived from adipocytes that are from obese mice contain miR-155 and carry this miRNA to macrophages. miR-142-3p and -5p are also packaged in EVs secreted from T cells and mediate the crosstalk between T cells and insulin target cells; (**b**) In cardiovascular diseases, macrophages and endothelial cells communicate with each other through miR-150 and miR-155 enriched in their EVs, respectively; (**c**) In cancers, tumor cells could secret EVs that transport miR-145, -222-3p or -103 to macrophages. Conversely, tumors cells receive miR-21 from macrophages through EVs. The arrows indicate the direction of EV transfer.

In addition to the cell-intrinsic modulation of miRNA-mediated regulation, intercellular communication through extracellular vesicles (EVs), which are complex structures composed of a lipid bilayer, may change miRNA functions by transferring miRNAs to recipient cells. Exosomes are small extracellular vesicles, 30–100 nm in diameter, found in blood and other body fluids, and are released by numerous cell types [128]. The critical roles of exosomes in the cell-to-cell dialogue of the immune system were first supported by the evidence that exosomes, which are enriched by major histocompatibility complex (MHC) class II molecules and are secreted from the Epstein–Barr virus (EBV)-transformed B cell lines, had the capacity to stimulate specific CD4^+^ T cell clones in vitro [129], and that tumor peptide-pulsed DC-derived exosomes were able to suppress the growth of established tumors in vivo [130]. After the first observation of miRNAs present in exosomes secreted by mast cells [131], several studies identified miRNA cargoes in exosomes secreted by immune cells, and conversely, exosomal miRNAs can be taken up by various immune cells [132,133,134,135]. By repressing target mRNAs, exosomally transferred miRNAs are able to influence the immune response of recipient cells. In addition to physiological roles, exosomal miRNAs are associated with disease progression since they appear to be affected by pathological conditions [136]. Given the frequent crosstalk between immune cells and the other cell types in chronic diseases, we will mainly discuss the findings in metabolic diseases, cardiovascular diseases, and cancers (Figure 2).

### 5.1. Metabolic Diseases

Approximately 500 miRNAs were present in adipose tissue macrophages-derived exosomes, which changed with obesity [137]. Adipose tissue macrophages-derived exosomes from obese mice induced glucose intolerance and insulin resistance in lean mice, which might be the consequence of enhanced miR-155 transportation to insulin target cell types where peroxisome proliferator-activated receptor gamma (PPARγ) is a target of miR-155 [137]. The exosome-mediated transfer of miR-142-3p, -142-5p, and -155 from T cells to pancreatic β cells promoted β cell apoptosis and induced cytokine and chemokine expression [138]. Notably, the inhibition of these three miRNAs using anti-miRs specifically protected β cells from T cell exosome-induced apoptosis, but not from the apoptosis induced by inflammatory cytokines. While specifically sequestering miR-142-3p, -142-5p, and -155 in β cells limited type 1 diabetes development in mice, it is not clear whether this is the result of endogenous miRNAs or from exogenous miRNAs transferred from T cell exosomes. Conversely, adipocyte-derived microvesicles from obese mice significantly enhanced inflammatory mediator expression in macrophages due to the delivery of miR-155 that targeted the suppressor of cytokine signaling 1 (SOCS1) [99]. This reprogrammed macrophage polarization may inhibit insulin signaling and glucose uptake in adipocytes.

### 5.2. Cardiovascular Diseases

Immune responses play an essential role in the development and progression of cardiovascular diseases, where immune cells often communicate with other cells. EVs secreted by human monocytes selectively packaged miR-150, which was further induced by pro-inflammatory challenges [139]. The incubation of microvascular endothelial cells with these EVs reduced the expression of the miR-150 target gene c-Myb, thereby enhancing cell migration. Notably, EVs isolated from the plasma of patients with atherosclerosis had similar effects [139]. Conversely, EVs secreted by endothelial cells exposed to oxLDL shifted the monocytes/macrophages balance from alternatively activated subtypes towards classically activated subtypes by delivering miR-155 [140]. 

Moreover, accumulating evidence suggests that a large number of miRNAs (such as miR-223) from cardiac stem cell-derived EVs exhibit cardioprotective characteristics by regulating the phenotype of recipient cells [141,142], although not all of which were immune cells. Since this review focuses on the immune system, we will only discuss the cases where immune cells are considered to be the recipient cells. Exosomes secreted by cardiosphere-derived cells induced macrophage polarization towards an anti-inflammatory and cardioprotective phenotype, mediated by miR-181b, and that repressed protein kinase C δ expression [143]. In addition to miRNAs, another class of non-coding RNAs, Y RNAs, was shown to be enriched in extracellular vesicles from cardiosphere-derived cells and played a cardioprotective role by promoting IL10 production in macrophages [144].

Microvesicles/exosomes derived from cardiomyocytes also play a vital role in cardiovascular diseases through transferring miRNAs to either cardiomyocytes [145] or other types of cells. For example, miR-92a and miR-195 from cardiomyocytes activated myofibroblast after myocardial infarction [146,147]. However, it is still not clear whether exosomal miRNAs mediate the communication between cardiomyocytes and immune cells. 

### 5.3. Cancers

Several kinds of tumor cells secrete EVs, which appear to have a similar effect on tumor-associated macrophages—promoting anti-inflammatory macrophage phenotypes and thereby, facilitating tumor growth. For example, EVs or exosomes derived from epithelial ovarian cancer, colorectal cancer, or lung cancers shifted macrophage phenotypes towards anti-inflammatory through transporting miR-222-3p, miR-145, and miR-103, respectively. While the role of miR-103 on tumor growth in vivo is still unknown, exosomal miR-222-3p and miR-145 have been shown to accelerate tumor progression [148,149,150]. Conversely, miR-21 was transferred from alternatively activated macrophages to gastric cancer cells through exosomes and inhibited the apoptosis of gastric cancer cells by regulating PTEN/phosphoinositide-3-kinase regulatory subunit 1 (Pik3r1, also known as PI3K)/Akt signaling and anti-apoptotic Bcl2 expression, leading to aggravated tumor growth [151].

Collectively, no matter the cell types, most, if not all findings, suggest that miRNAs are selectively packaged in EVs; however, the mechanisms of miRNA cargo selection in EVs are still not clear. Once this issue is addressed, exosomal miRNA-based therapy might be a safe and effective approach to treat human diseases. 

## 6. Conclusions and Future Perspectives

While the key role of miRNA-mediated gene regulation has been established in the immune system, there are still many unresolved questions regarding how immune cells modulate this process during immune responses. The transcription factors of most miRNAs are yet to be identified, which will greatly advance our understanding of changes in miRNA profiles in activated immune cells challenged by various stimuli. The precise and specific effects of RBPs on miRNA biogenesis and how these proteins are regulated need to be investigated in the development and function of the immune system, as well as in pathological processes. Moreover, further studies are needed to reveal how miRNAs select their targets, depending on the cell type and immune microenvironment. In addition, the factors determining the specificity of miRNAs packaged in EVs derived from immune cells are still unknown. The answers to these questions should lead to new opportunities for drug discovery based on the upstream regulators of miRNA pathways.

## Figures and Tables

**Table 1:** Transcription factors (TFs) and epigenetic modifications regulating microRNAs (miRNA) transcription in immune cells.

Cell Type	TF or Epigenetic Modification	Regulated miRNA	Correlation	References
Transcription factor				
Granulocyte	PU.1	miR-223	Positive	[34]
Hematopoietic stem cell		miR-23a	Positive	[36,37]
Dendritic cell Macrophage		miR-142-3p	Positive	[38] [39]
THP-1	NF-κB	miR-146a	Positive	[44]
THP-1		miR-155	Positive	[47,49]
Macrophage		miR-33, miR-33*	Positive	[50]
Dendritic cell		miR-22	Positive	[51]
Macrophage	Ets2	miR-155	Positive	[47]
Treg cell	Foxp3	miR-155	Positive	[52]
Tfh	Bcl-6	miR-17, miR-20a	Negative	[53]
CD8^+^ T cell	NFAT	miR-31	Positive	[54]
Epigenetic modification				
Macrophage	DNA methylation	miR-495	Negative	[55]
Macrophage	Histone demethylation	miR-146a	Negative	[56]
CD4^+^ T cell	DNA methylation	miR-21	Negative	[57]
B cell	Histone acetylation	miR-155, miR-181b	Positive	[58]
